# Abnormal Insular Dynamic Functional Connectivity and Its Relation to Social Dysfunctioning in Children With Attention Deficit/Hyperactivity Disorder

**DOI:** 10.3389/fnins.2022.890596

**Published:** 2022-05-31

**Authors:** Ahmed Ameen Fateh, Wenxian Huang, Tong Mo, Xiaoyu Wang, Yi Luo, Binrang Yang, Abla Smahi, Diangang Fang, Linlin Zhang, Xianlei Meng, Hongwu Zeng

**Affiliations:** ^1^Department of Radiology, Shenzhen Children's Hospital, Shenzhen, China; ^2^Children's Healthcare, Mental Health Center, Shenzhen Children's Hospital, Shenzhen, China; ^3^Shenzhen Graduate School, Peking University, Shenzhen, China

**Keywords:** attention deficit hyperactivity disorder, dynamic functional connectivity, insula, rs-fMRI, social dysfunction

## Abstract

Anomalies in large-scale cognitive control networks impacting social attention abilities are hypothesized to be the cause of attention deficit hyperactivity disorder (ADHD). The precise nature of abnormal brain functional connectivity (FC) dynamics including other regions, on the other hand, is unknown. The concept that insular dynamic FC (dFC) among distinct brain regions is dysregulated in children with ADHD was evaluated using Insular subregions, and we studied how these dysregulations lead to social dysfunctioning. Data from 30 children with ADHD and 28 healthy controls (HCs) were evaluated using dynamic resting state functional magnetic resonance imaging (rs-fMRI). We evaluated the dFC within six subdivisions, namely both left and right dorsal anterior insula (dAI), ventral anterior insula (vAI), and posterior insula (PI). Using the insular sub-regions as seeds, we performed group comparison between the two groups. To do so, two sample *t*-tests were used, followed by *post-hoc t*-tests. Compared to the HCs, patients with ADHD exhibited decreased dFC values between right dAI and the left middle frontal gyrus, left postcentral gyrus and right of cerebellum crus, respectively. Results also showed a decreased dFC between left dAI and thalamus, left vAI and left precuneus and left PI with temporal pole. From the standpoint of the dynamic functional connectivity of insular subregions, our findings add to the growing body of evidence on brain dysfunction in ADHD. This research adds to our understanding of the neurocognitive mechanisms behind social functioning deficits in ADHD. Future ADHD research could benefit from merging the dFC approach with task-related fMRI and non-invasive brain stimulation, which could aid in the diagnosis and treatment of the disorder.

## 1. Introduction

Attention Deficit Hyperactivity Disorder (ADHD) is the most commonly diagnosed condition in children, characterized by age-inappropriate problems like inattention, impulsivity, and hyperactivity (Thomas et al., 2015; Sayal et al., 2018). ADHD is therefore related to cognitive, academic, familial, and occupational problems (Usami, 2016). Social functioning is also directly impacted by ADHD. This might be manifested as peer rejection and disagreements. Social dysfunction may negatively affect the short- and long-term prognosis of ADHD youngsters. The activities that encourage social inadequacies are directly linked to ADHD diagnosis in some infants. While children with ADHD crave social interaction, they often struggle to adapt their behavior to their environment due to the nasty, angry tone of their interactions as well as their hyperactive/impulsive behavior. This implicates rule infractions, aggressive and dominating behavior, and physical and verbal animosity. It also includes agitation and intrusion, which are often inappropriate and difficult to remedy (Lahey et al., 2005).

ADHD is linked to functional deficits in the cognitive, academic, familial, and occupational areas of everyday life (Usami, 2016). Social functioning is another crucial aspect of ADHD that is directly affected. This might present itself as peer rejection and disputes with other children and adults. Social dysfunction may have a significant negative impact on the short- and long-term prognosis of children with ADHD. The practices that promote social deficiencies may be a direct result of diagnosing symptoms of ADHD in at least some infants. Some of ADHD's DSM-IV criteria, such as “interrupting or intruding on others,” even explicitly relate to poor social conduct (Lahey et al., 2005). Generally, the combination of hyperactivity, impulsivity, and inattention is likely to affect social behavior. Although children with ADHD have a strong desire to interact with others, they typically struggle to adapt their attitude to their surroundings. Two behavioral characteristics are typically linked to social difficulties in children with ADHD, namely the unpleasant, hostile tone of their interactions, as well as their hyperactive/impulsive behavior. Rule violations, antagonistic and dominating behavior, and the use of physical and verbal hostility are examples of the first aspect. These actions may pose a direct threat to others, and they have been proven to be substantial predictors of negative peer nominations in both ADHD and non-ADHD children. Examples of the second aspect comprise restless and invasive conduct, which is frequently inappropriate in the current setting and difficult to remedy (Lahey et al., 2005).

The orbitofrontal cortex (OFC), the amygdala, and the temporal cortex (mostly the superior temporal sulcus-STS) were found to be primary elements of the so called “social brain,” in the early 1990s (Brothers, 1990). Afterwards, other areas like the medial prefrontal cortex (mPFC) and the anterior cingulate cortex (ACC), were shown to be primary for social functioning and were therefore included with the initial core (Frith and Frith, 2006; Bickart et al., 2014). Modern definitions of social brain usually incorporate dynamic and hierarchical structure of circuitry entangled in elementary constructs of more automated systems like the identification of socially significant stimuli as well as relatively overlapping circuitry implicated in higher-order operations of the psychological condition. For instance, feelings such as disgust or anger were basically associated with the aversion network where the insula is key component (Buckholtz et al., 2008). This implicates that the insula, among other brain regions in the aversion network, is mediated in aversive behaviors such as avoiding strangers that are not trustworthy. Studies have also demonstrated the implication of the insula in the "social decision making" that enables the selection of flexible behavioral responses to others (Rogers-Carter and Christianson, 2019). More precisely, the insular cortex is anatomically located to connect integrated social sensory cues to the social decision making network, resulting in flexible and adaptive behavioral outcomes to social and emotional stimuli. In line with these findings, Belfi et al. (2015) suggested that subjects with lesion on the insula had aberrant trust expressions. During a trust game, when acting as an investor, these people behaved benevolently (showing misguided trust), and when acting as a trustee, they acted malevolently (infringing their partner's trust). Although the topic of attention, play and social behavior in children with ADHD has been studied for years now, yet, it is unfortunate that tackling the role of the insula in the social dysfunctioning in children with ADHD is still scarce. Large body of research barely mentioned the insula as part of different networks related to social functioning, especially with regard to neuroimaging-based investigations.

The majority of neuroimaging research indicates static brain networks across the course of an fMRI session. These networks show functional connections and interactions between distinct cortical and subcortical brain regions during task execution or at rest. However, because activity in static networks does not clearly display changes that occur over short periods of time during an fMRI scan, dynamic reconfiguration-based methods to discover the modular architectures of changing networks are becoming more popular and the so called dFC was introduced. By partitioning fMRI images into time windows, the interconnections between brain regions can be better understood. Dynamic reconfigurations are more sensitive and able to detect more changes in human brain activity than static reconfigurations (Patil et al., 2021). whether static or dynamic, at rest or task-based, accurately identifying the altered functional connectivity generated by ADHD or any other specific disorder is a critical endeavor that may reveal the disorder's causative factors. Both in childhood and adulthood, imaging studies have revealed structural and functional abnormalities in the brains of ADHD patients. Many evidence from fMRI studies strongly suggest that biomarkers and alterations in interactions within and between different brain connectivity may contribute to the disruption of normal brain functions and cognitive performance, leading to fluctuations in attention in patients with ADHD (Sonuga-Barke and Castellanos, 2007; Shappell et al., 2021). Functional impairments in fronto-cortical and fronto-subcortical networks are basic deficiencies in both children and adults with ADHD, according to Rubia et al. (2014). Consistent with these findings, Guo et al. (2020) questioned the consistency of ADHD diagnosis from childhood to maturity, as well as the similarities and differences in abnormal functional connectivities (FCs) across ADHD children and adults. To put it another way, they looked at clinical changes and pathophysiological continuity in ADHD patients from childhood to adulthood. On the other hand, a thorough research in the literature yielded to very few researches addressing dFC in ADHD. For instance, Ahmadi et al. (2021) revealed that subtypes of ADHD have generalized anomalies in static FC and dFC between large-scale resting state networks, encompassing cortical and subcortical areas, when compared to typically developing youngsters. They came to the conclusion that dynamic changes in brain FC may better help to explain the pathophysiology of ADHD. Sun et al. (2021) suggested state-dependent dynamic changes in large-scale brain connections and network topologies in ADHD. Yang et al. (2021) found that children with ADHD have more unstable dFC of the amygdala subregions, which may impact their cognitive skills. As a result, it should be indicated that to ensure successful diagnosis, therapy, and prevention, it is critical to research the sophisticated mechanisms underlying ADHD, as well as the functional deficits of the diseased brain. rs-fMRI is one such a non-invasive and safe method of detecting spontaneous brain activity (Lu et al., 2018). Over the last three decades, numerous studies have been conducted to investigate potential imaging changes and biomarkers of ADHD. However, no significant findings were yield to study the dFC of the insula in children with ADHD, and its contribution to social dysfunction.

From a neurobiologic standpoint, ADHD is increasingly being recognized as a disorder resulting from disruptions in large-scale brain networks. Extant studies of brain's FC in ADHD, however, have provided inconsistent outcomes, with some research suggesting hyper- and hypoconnectivity with respect to neurotypical controls and others providing null findings, mostly between the same brain networks, likely due to weak theoretical models, inadequate quantitative approaches, and variation in protocols and measures across data collection silos. Importantly, little is understood about the dynamics of brain connectivity in ADHD, because earlier research assumed that functional linkages across brain regions or networks were stationary. Aiming to overcome these challenges, this study investigated Insular subregions to test the hypothesis that insular dFC among different brain regions is impaired in children with ADHD, and these impairments may play a role in social dysfunction. Furthermore, the neural biomarkers found in children with ADHD were analyzed to see if they might be used as group-level features to distinguish patients with ADHD from HCs. The findings of this study could provide new imaging-based insights that can assist explain the clinical manifestations of ADHD and improve our understanding of the brain mechanism behind its symptoms in the pathway to ADHD.

## 2. Materials and Methods

### 2.1. Participants and Measures

Shenzhen Children's Hospital provided data with a total of 30 ADHD boys aged between 7 and 10 years and 28 HCs having the same age range. Two experienced psychiatrists assessed all of the patients to ensure that they met the diagnostic criteria for ADHD based on clinical interviews that followed the Diagnostic and Statistical Manual of Mental Disorders, Fourth Edition. The participants and their parents were interviewed using the Schedule for Affective Disorders and Schizophrenia for School-Age Children-Present and Lifetime Version interview (K-SADS-PL; Kaufman et al., 1997). ADHD diagnoses were based on the Diagnostic and Statistical Manual of mental disorders-fourth edition (DSM IV) (Association, 2013). Clinically-referred children who satisfied the DSM-IV criteria (either mixed, mainly inattentive, or predominantly hyperactive/impulsive subtype) were included in this study.

For each ADHD patient, parents, teachers, and other people who are in charge of caring for the kid were asked about the child's behaviors and conducts in various settings, such as at home, school, or with peers. The Conners-3 parent/teacher ratings (Conners, 2008) was used to evaluate ADHD symptoms and associated issues such as disruptive behavior and learning difficulties. The used lengthy Conner-3 version has 105/111 items (parent/teacher) that are graded on a 4-point Likert-scale from 0 (never) to 3 (very much/very frequently). The Conners-3 comprises scales such as hyperactivity/impulsivity, inattention, learning problems, executive functions, aggression, peer relations (content scales); DSM IV-inattention and hyperactivity/impulsivity, DSM IV-conduct disorder, DSM IV-oppositional defiant disorder (symptom scales); ADHD index, Global index. We obtained an internal consistency Cronbach's α (Christiansen et al., 2016) of 0.84 for the content scales and α = 0.80 for the symptom scales of the Conners-3 parent rating scale. Children with ADHD who showed persistent patterns of inattention and/or hyperactivity-impulsivity that affect their functioning and development for at least 6 months were diagnosed. Accordingly, healthcare providers examined symptoms of inattention such as: (1) failing to pay close attention to details or making thoughtless blunders, in schoolwork, (2) frequently struggling to maintain focus on chores or recreational activities, (3) frequently ignoring instructions and directions and failing to complete homework or chores (e.g., loses focus, side-tracked), (4) having a hard time keeping track of tasks and activities, (5) during regular activities, the kid is prone to forgetfulness and distraction, (6) frequently misplaces items required for chores and activities (e.g., school materials, pencils, books, tools, eyeglasses), (7) frequently avoids, hates, or is hesitant to accomplish tasks that demand sustained mental effort (such as schoolwork or homework). Symptoms related to hyperactivity and impulsivity were also examined such as: (1) being unable to play or participate in leisure activities in a peaceful manner, (2) talking excessively, (3) having difficulty waiting for their turn, (3) disturbs or invades the privacy of others (e.g., butts into conversations or games). The cognitive function was assessed using the Stroop Color and Word Test (SCWT) (Lee and Chan, 2000) which indeed has effect on the working memory that can, in turn, have behavioral consequences similar to those of externally perceived stimuli. As for the assessment of social functioning, there were specific questions about the number of close friends, the contact with them. Ratings were made on a 4-point scale (less than one, 1–2, 3–4, 5 or more). The quality of ADHD children's relationships with friends and their reactions to family members visits was also evaluated using a questionnaire of a 5-point scale [from 0 (no contact/reaction at all) to 5 (very well)].

The K-SADS-PL was used to consult the children in HCs group, as well as their parents, to check that they did not fulfill the diagnostic criteria for ADHD or any other mental illnesses. Normal eyesight and hearing were also required, as well as a Full-Scale Intelligence Quotient (FSIQ) ≥70 calculated using the Wechsler Intelligence Scale for Children, Fourth Edition. Participants with current or previous psychological illnesses, major physical disorders, neurological disorders, or brain injuries were not allowed to participate in this study. The Shenzhen Children's Hospital Medical Research Ethics Committee gave their approval to this study. All of the children agreed to take part in this study, and their parents gave signed informed consent.

### 2.2. rs-fMRI Data Acquisition

The rs-fMRI data for all participants were obtained using a 3.0-T system scanner (Siemens Magnetom Skyra) at the Radiology Department of Shenzhen Children's Hospital, Shenzhen, China. The rs-fMRI data were acquired using echo-planar imaging (EPI) sequence with the following parameters: repetition time (TR) = 2, 000*ms*; echo time = 30*ms*; flip angle = 90°; matrix size = 64 × 64; 32 axial slices; field of view = 24 × 24*cm*^2^; slice thickness = 3*mm* and no gap. Structure 3D-MPRAGE; T1 Repetition Time [TR, ms] = 2,300 ms, Echo Time [TE, ms] = 2.26; Number of Averages = 1.0, Slice Thickness = 1.0*mm*, Field of View (FOV) = 256*mm*.

### 2.3. Data Pre-processing

The DPABI toolkit (Yan et al., 2016) was used to preprocess the data. Because of the volatility of the initial magnetic resonance imaging signal and the participants' adaption to the experimental setup, the first 10 volumes were eliminated. The remaining 220 volumes were first realigned to correct for head-motion before being corrected by the acquisition time delay among different slices. Under the head motion criterion of ±3*mm* and no participant was excluded. The pictures were then normalized and resampled into a voxel size of 3 × 3 × 3*mm*^3^ utilizing an uniform segmentation of anatomical images. The following three steps were engaged in normalization: 1) Each participant's T1 structural images were co-registered to their corresponding functional images; 2) Co-registered T1 images were segmented into gray matter, white matter, and cerebrospinal fluid using transformation parameters that indicated transformation from subject native space to standard Montreal Neurological Institute (MNI) space; 3) Functional images were finally transformed into the standard space using transformation p. Additional regression was applied to nuisance factors, such as 24 head movement parameters, global signal, white matter signal, and cerebrospinal fluid signal, to adjust for physiological noise, such as motion and cardiac and respiratory cycles. Following that, the data were linearly detrended, filtered at 0.01–0.08 Hz, and smoothed with a 6*mm* full-width-at-half-maximum Gaussian kernel.

### 2.4. Head Motion

The mean framewise displacement (FD) created during the scanning process was removed using Jenkinson's relative root-mean-square technique (Jenkinson et al., 2002). To evaluate the voxel-wise motion differences between the two groups, the mean FD (Jenkinson) was determined. The mean FD did not change substantially between the ADHD and HC groups (*p* < 0.6).

### 2.5. Static Functional Connectivity Analysis

Seed areas were chosen based on the presence of social dysfunctioning-related anomalies in right and letf dAI, vAI, and PI in FC. The seeds were obtained using cluster analysis, in agreement with earlier literature (Deen et al., 2011), in which the insula was subdivided based on FC pattern clustering. We were primarily interested in the rdAI and rvAI regions, which have been linked to attention and emotion, as well as an outwardly and inwardly oriented system, respectively (Touroutoglou et al., 2012). The right and left hemispheres of the dAI, vAI, and PI were then transformed to MNI 152 standard brain (3-mm resolution) and used as seed regions in the FC analysis in HC and ADHD patients. We used the REST toolbox (http://restfmri.net/forum/index.php) to perform seed-based FC studies to evaluate the aberrant sFC of seed regions in HC and ADHD. Between the mean time course of each seed region and the time course of all other voxels in the entire brain, Pearson's correlation coefficient was derived. To increase the Gaussianity of their distribution, the resulting r maps were turned into z maps using Fisher's r-to-z transformation. We obtained z-score maps for each subject that represented the sFC of the right and left of dAI, vAI, and PI.

### 2.6. Dynamic Functional Connectivity Analysis

Using the DynamicBC toolbox (Liao et al., 2014), the sliding window method was used to analyze the dFC for each participant. According to previous research, the window length is an open but important parameter in sliding window based resting state dynamic computation (Fateh et al., 2020; Li et al., 2020; Yang et al., 2021). This approach can calculate the time-varying covariance of interregional neural signals, which is the variance of dFC, and reveal the temporal aspects of FC during the full scan period. The sliding window method uses the window length as a crucial parameter.The minimum window length should not be smaller than 1/fmin, as per Leonardi and van de Ville (Leonardi and Van De Ville, 2015), because a very short window length may generate spurious fluctuations. In addition, the fmin represents the time courses' minimal frequency. The dynamic properties of the time series would be made unobservable if the window length was too long. We chose a window length of 50 TRs (i.e., 100s) and a step size of 1 TR because a window length of 50 TR was proposed to maximize the balance between recording a fast altering dynamic relationship and producing credible estimations of the correlations between regions (Liao et al., 2018) (i.e., 2s). In the validation analyses that followed, other window lengths and step sizes were also evaluated. Liao et al. (2018) computed the Fisher's z-transformed Pearson's correlation coefficient between the average time series of each seed region and the remaining voxels in the whole brain in each window. As a result, each participant received a set of sliding-window correlation maps. Calculating standard deviation values at each voxel across sliding-windows was used to estimate the dFC.

### 2.7. Statistical Analyses

**Figure 1:**
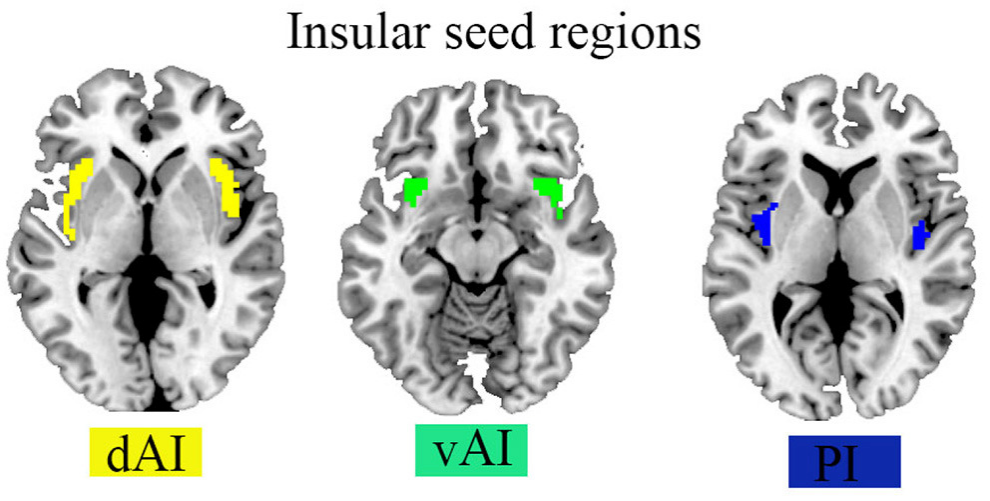
Seed regions of the insula in the Brian.

To see if there was a difference in the dFC of the insular subregion between HCs and ADHD patients, a two-sample *t*-test model was used. Confounding factors such as the mean FD, age, gender, and grade were regressed out. T-statistic images were transformed to z-statistic images, and then thresholder using clusters identified by a z value of >2.3 and a cluster-level thresholder *p*-value of 0.05, corrected for whole-brain multiple comparison correction using Gaussian random field theory. The regions of interest (ROIs) for the *post-hoc* analysis were chosen from the survivors' brain clusters. The data were corrected by multiple comparisons using Gaussian random field theory (GRF, voxel-wise *p* < 0.001, cluster-wise *p* < 0.05, two-tailed) and the dynamic R-fMRI indices and voxel-wise concordance were compared using a two-sample *t*-test. On these ROIs, a two-tailed, two-sample *t*-test was used to evaluate the differences between two groups (HC vs. ADHD). The statistical significance level is *p* < 0.05/6. (Bonferroni correction). Brain regions data are summarized in Table 1 and the positions of the Insular sub-regions are depicted in Figure 1.

**Table 1:** Brain clusters showing significant effects in the dFC of insular subregions.

**Seed region**	**Brain regions**	**Cluster size**	**Z score**	**M N I**	**ADHD(*n* = 30)**	**HC(*n* = 28)**
		**Voxels**		**X Y Z**	**M ±SD**	**M ±SD**
Right dAI	Left middle frontal gyrus	80	–5.76	–33 9 36	0.02 ± 0.01	0.05 ± 0.01
Right dAI	Left postcentral gyrus	85	–5.02	–48 –33 63	0.02 ± 0.009	0.05± 0.019
Right vAI	Right cerebelum_crus	91	–5.54	33 –75 –48	–0.13 ± 0.14	–0.001± 0.12
Left dAI	Left thalamus	68	–7.75	–3 –15 3	0.02 ± 0.012	0.05± 0.019
Left vAI	Left precuneus	79	8.1	–42 –60 0	–0.09 ± 0.15	0.02 ± 0.14
Left PI	Right temporal pole	65	–6.27	43 20 –22	0.02 ± 0.0.01	0.05 ± 0.016

*SD, Standard Deviation; M, Mean value; dAI, dorsal Anterior Insula; vAI, Ventral Anterior Insula; PI, Posterior Insula*.

### 2.8. Validation Analysis

We performed validation analysis for several sliding window lengths besides 50 TR to corroborate our findings of dFC variability derived from 50 TR lengths of the sliding window. As a consequence, we recalculated the primary dFC results with the other two window lengths (30TR and 80TR).

## 3. Results

### 3.1. Demographic and Clinical Information

The demographic and clinical characteristics of the ADHD and HC groups were listed in Table 2. No differences in sex and mean FD were detected between the two groups. We found a large differences in IQ, working memory and learning problems between the two groups, in a way that these variables were lower in ADHD patients compared to HCs. For social functioning, children with ADHD had a significantly lower number of social contacts, and a poorer quality of social contacts with family members compared to the HCs group. It was also shown that ADHD patients had more problems with social relations with their peers although the effects were marginally significant (i.e., *p* < 0.1).

**Table 2:** Demographic and clinical information.

**Variables**	**HC (*n* = 28)**	**ADHD (*n* = 30)**	***p*-values**
Age, mean ± SD	8.6 ± 0.97	8.6 ± 0.56	0.11^a^
Sex (male)	28	30	
Grade, mean ± SD	3 ± 0.83	2.6 ± 0.56	0.02^a^
FD, mean ± SD	0.05 ± 0.02	0.06 ± 0.02	0.6^a^
IQ scores, mean ± SD	108.6 ± 10.81	84.3 ± 9.37	<0.001^a^
Working memory, mean ± SD	19.5 ± 3.10	9.95 ± 2.11	<0.001^a^
Behavioral problems, mean ± SD	1.12 ± 0.45	0.50 ± 0.40	<0.001^a^
Anxiety score, mean ± SD	0.54 ± 0.35	0.12 ± 0.10	<0.01^a^
Learning problems, mean ± SD	1.15 ± 0.5	0.45 ± 0.30	<0.01^a^
Psychosomatic disorder, mean ± SD	0.21 ± 0.18	0.12 ± 0.30	0.51^a^
IS(time), mean ± SD	18.2 ± 8.5	9 ± 3.5	<0.01^a^
Number of social contacts	3.2± 0.80	2.98± 0.82	<0.1^a^
Quality of social contacts-family	3.45 ± 1.07	3.34 ±1.04	<0.1^a^
Quality of social contacts-friends	3.78 ± 1.50	4.10 ± 1.28	
Problems with social relations with their peers	3.00 ± 2.19	3.04 ± 2.80	<0.1^a^

*HC, healthy control; ADHD, Attention deficit hyperactivity; FD, frame-wise displacement; SD, Standard deviation; a, Two-samplet-test; IS, interference score*.

### 3.2. Differences of the dFC in the Right dAI, vAI and PI Among ADHD, and HCs

**Figure 2:**
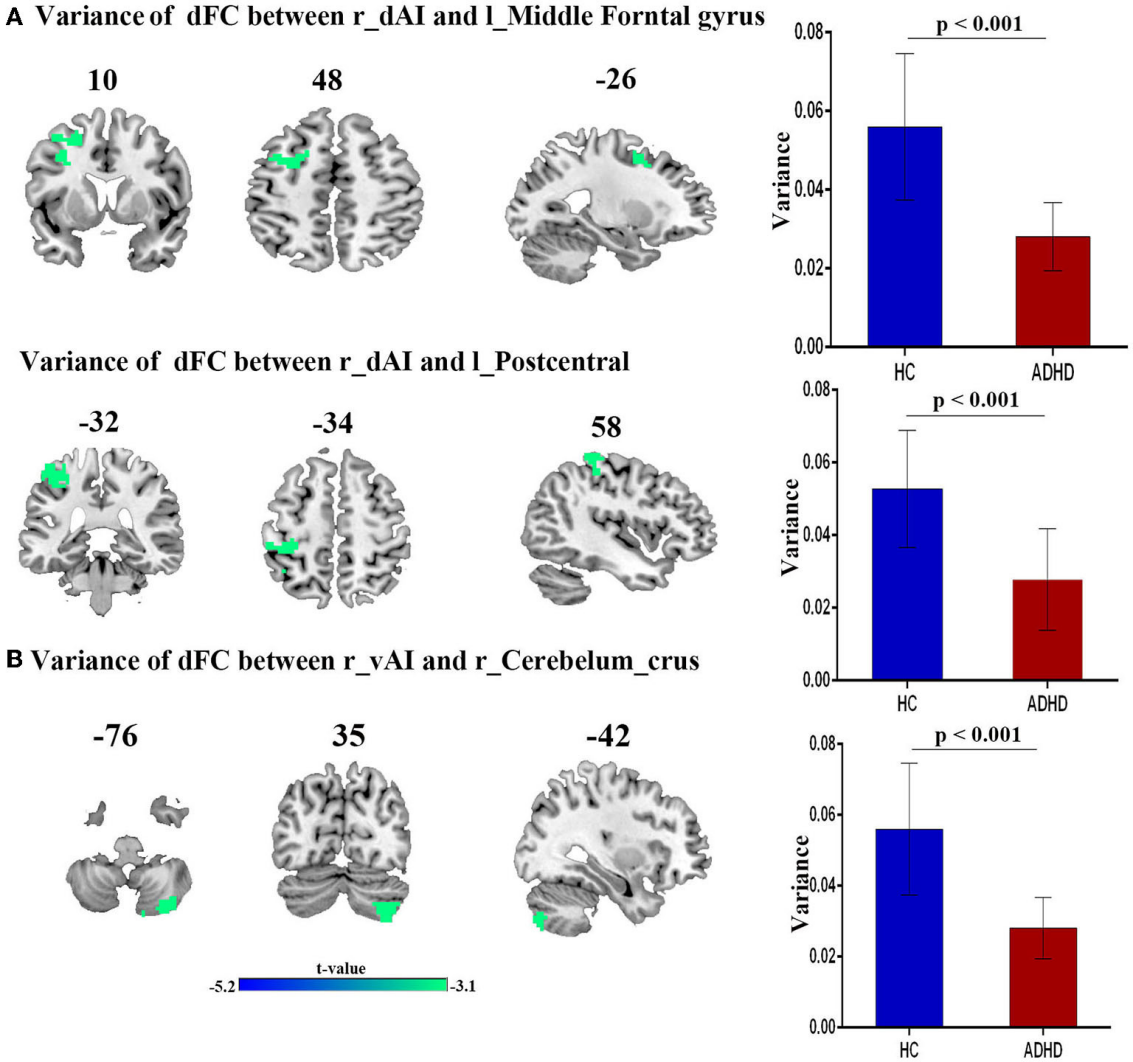
The variance of dFC between the right insula and other brain regions, obtained by comparing HCs and ADHD groups, using two sample *t*-test; **(A)** Between right dAI and Left Middle frontal gyrus (first row) and Left postcentral gyrus (second row). **(B)** Between right vAI and right cerebelum curs.

Compared with HCs, patients with ADHD showed significantly decreased dFC between right dAI with left middle frontal gyrus and left postcentral gyrus and between right vAI with right cerebellum crus. No significance was found in the PI. Moreover, no increased dFC has been obtained between the two group. Details regarding information on differences of dFC in between-group are introduced in Figure 2 and Table 2.

### 3.3. Differences of the dFC in the Left dAI, vAI and PI Among ADHD, and HC

**Figure 3:**
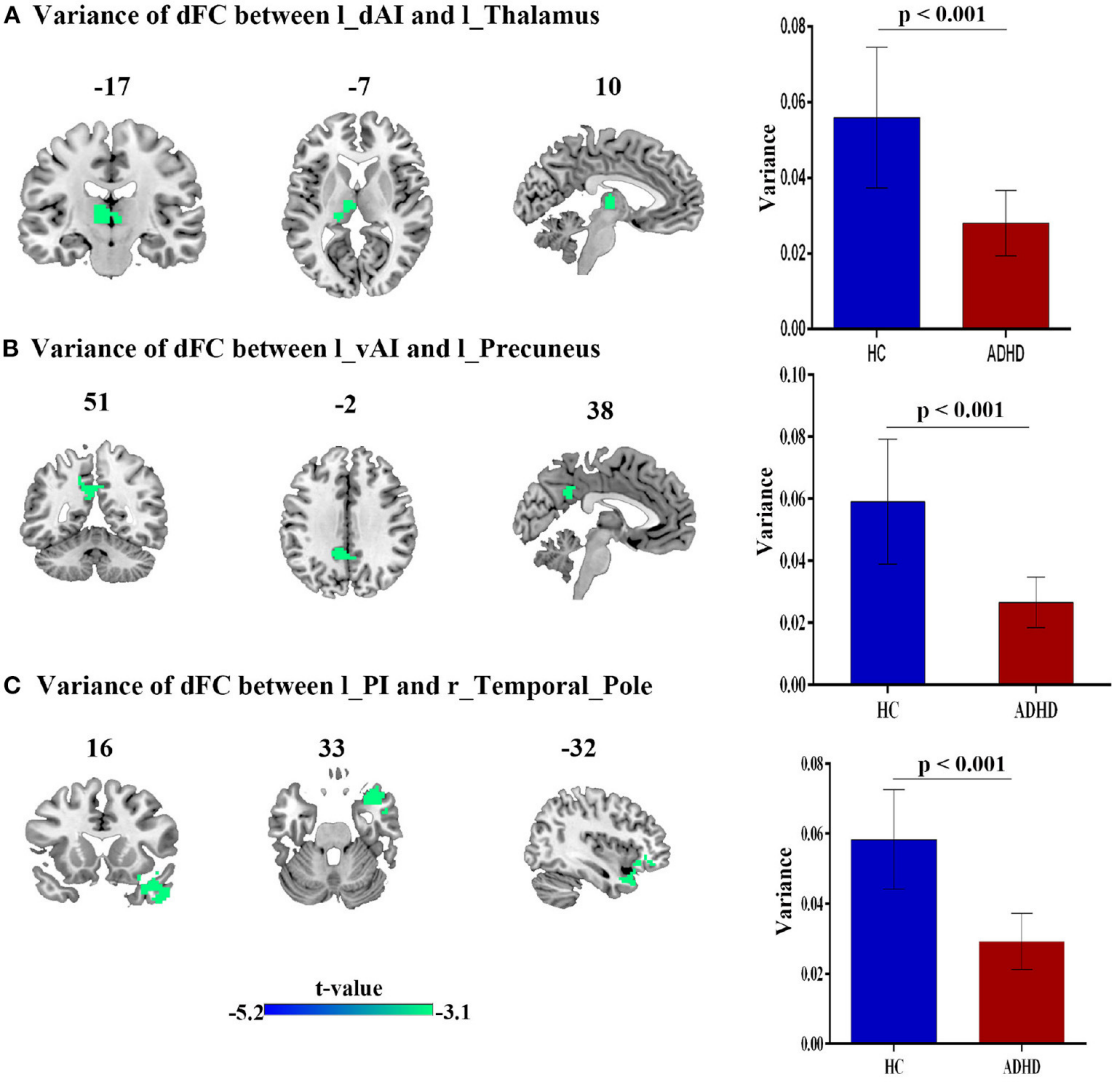
The variance of dFC between the left insula and other brain regions, obtained by comparing HCs and ADHD groups, using two sample *t*-test; **(A)** Between left dAI and Left thalamus. **(B)** Between left vAI and left precuneus, **(C)** between left PI and right temporal_pole_mid.

Compared with HCs, patients with ADHD showed significantly decreased dFC between dAI, vAI and PI and left thalamus, left precuneus and right temporal pole, respectively. No increased dFC has been detected between the two group. Details pertaining to the results of between-group differences in dFC of left subregions of insula is presented in Figure 3 and Table 2.

### 3.4. Validation Analyses

To verify our findings of dFC in insular subregions variability obtained from sliding-window length of 50 TRs (100s), we performed auxiliary analyses with different sliding window lengths. We recalculated the main results by using two other window lengths (30 TR and 80 TRs) were similar to the main results of 50 TR in our study.The corresponding results are shown in the Supplementary Materials. All validation analysis results are presented in Supplementary Figures S1–S4.

## 4. Discussion

Impaired attention, impulsivity and hyperactivity are the solely hallmarks in ADHD. These symptoms are directly related to social dysfunctioning that mostly affects the daily life of ADHD patients, especially kids. Despite its importance to the neuroimaging research community, few studies about dFC in children and adolescents with ADHD have been published so far. Although there are no clinically accurate biomarkers for the diagnosis of ADHD, this study investigated the insula dFC with other brain regions in the hope that such research can promote to the discovery of numerous viable candidate biomarkers, especially those associated to social dysfunctioning. Our findings support the hypothesis that the insular dFC with distinct brain regions is altered, and these deficits may be implicated in social dysfunction. Consistent with our hypothesis, compared to HCs, patients with ADHD showed decreased dFC values between right dAI and the left frontal_mid gyrus, left postcentral gyrus and the right of cerebellum crus. Results also indicated a decreased dFC between left dAI and thalamus, left vAI and left precuneus and left PI with temporal pole mid.

Social dysfunctioning is a broad term that is manifested by various neuropsychiatric disorders. To date, the majority of studies investigating social functioning have relied on self-report, questionnaire-based measures of social function (Hodgetts et al., 2017). In this study, with regard to ADHD, during diagnosis testing, we opted for a number of aspects (i.e., cognitive and executive functioning, working memory, learning problems, and anxiety-related symptoms and other measures; namely the number of social contacts, the quality of social contacts for both family and friends and the problems with social relations with their peers) depending on the available participants' data. These aspects entail other related concepts such as emotion regulation and attention orientation. The current findings, as well as those from earlier studies (Biederman et al., 1993), suggest that children with ADHD frequently experience issues with social relationships. Due to their behavioral problems (e.g., not following the rules when playing a game), these children may not have the same opportunities to make friends. Other impaired functional connectivity that are possibly associated with social dysfunctioning are discussed below considering our dFC's findings. We also found a considerable differences in IQ, working memory and learning problems between the two groups. Many studies have demonstrated that both IQ and working memory are related to learning in a sense that these IQ and working memory would predict reading, writing, and math skills in children (Alloway and Copello, 2013). Our findings are consistent with other existing studies in the literature (Rohrer-Baumgartner et al., 2014) that found in children with below median IQ-score, a larger number of ADHD symptoms were more likely to be accompanied by reports of lower expressive language skills. One possible reason for such lower scores and their implications to the lower expressive language skills is due to impaired (decreased) dFC between the Insula and the frontal middle gyrus since these two regions have been involved in language, self expression and learning. More related interpretations are presented below.

The insula (or insular cortex) is a thin ribbon of gray matter tissue that lies just deep to the lateral brain surface, separating the temporal lobe from the inferior parietal cortex (Broder and Preston, 2011). Taste, visceral sensation, and autonomic control are only a few of the homeostatic activities pertaining to basic survival needs that involve the insula. It was also proved that the insula regulates the sympathetic and parasympathetic nervous systems, which control autonomic activities (Bud Craig, 2009). According to functional connectivity studies and in line with our study, the human insula has at least three different segments (Nomi et al., 2016). A dorsal anterior insula (dAI) subdivision with connections to the frontal, anterior cingulate, and parietal areas is participated in cognitive control processes; a ventral anterior insula (vAI) subdivision has connections to limbic areas and is involved in affective processes; and a mid-posterior insula (PI) subdivision has connections to brain regions for sensorimotor processing (Cereda et al., 2002). Based on our dFC analysis, while studying time-varying patterns of interactions between insular subdivisions and other brain regions, we found that the dAI has more changeable connections than the other insular segments. This is consistent with previous research indicating the dAI's functional "diversity," which is engaged across numerous task domains (Penfield and Faulk, 1955; Cereda et al., 2002). On the other hand, recent functional imaging investigations have found that the left or right lateralization of emotional processing is influenced by stimulus valence (positive or negative emotions) (Harrington, 1995), behavior (approach/withdrawal) (Davidson et al., 1990), and subjective state (perception/experience) (Peelen et al., 2010). In regard to the lateralization of the human insula, our findings revealed the following insights: (1) stronger dFC of the insula in the left hemisphere than the right, which was manifested among all subdivisions (2) heterogeneous connectivity between insula subdivisions' profiles. In accordance with these findings, a number of lesion case studies elucidated the role of left insula in executive set-switching (executive functioning) which is mainly associated with ADHD. For instance, Varjačić et al. (2018) suggested support for the role of the left insular cortex in flexible attention switching among stroke survivors. Markostamou et al. (2015) studied the case of a woman with an acute left anterior insular infarction that led to executive (word and design fluency, mental flexibility, sustained attention, inhibitory control) but not language, visuoperceptual, or memory deficits. Conflicting with these findings, by considering the the way we relate language to our interpersonal relationships, while some functional imaging studies reported greater activation in the left insula in equal bilingual young adults (Chee et al., 2004), others demonstrated the brain's ability to sustain proper language without the insula (Duffau et al., 2001). On the other hand, regarding our second finding which supports many relevant recent researches proposing a tripartite organization rather than the traditional anterior-posterior dichotomy. Nomi et al. (2018) elucidated that the functional profiles of the insular subdivisions are both unique and overlapping.

Our decreased dFC results were compatible with other studies (Wang et al., 2020) that found significant decreases in both functional connectivity and global network efficiency. This decrease may correspond to either patients' insula incapability to integrate external sensory information with cognitive abilities including supervisory attentional control (Cieslik et al., 2015) and interior emotion or to the small size of the data sample. The former inference is due to the anatomy of the nervous system whereby the thalamus takes information from “homeostatic afferent” sensory pathways and transfers it to the insula, which then sends it to a number of limbic-related regions, including the amygdala.

Another key result of this study is the alteration of dFC between left AI and left thalamus. Consistent with our findings, in regard with social dysfunctioning, the processing of information relevant to gustatory, visceral, and autonomic functions, and even salient information and emotion regulation, is underpinned by connections between the thalamus and the anterior insula (Ghaziri et al., 2018). A decreased FC with limbic regions such as the amygdala, hippocampus, thalamus, and insula in people with subclinical anxiety were reported in Scheinost et al. (2013) and in ADHD adolescents (Rubia et al., 2019). Mills et al. (2012), in turn, also suggested a corticostriatal-thalamic connectivity changes in children with ADHD and they then discussed the relation of these results to patients' working memory ability. Working memory is critical for reasoning, decision-making, and behavior guidance and it is among the core difficulties especially for students with ADHD. We continually handle social cognitive information, whether it's keeping track of friends' viewpoints during conversation, a roomful of colleagues' beliefs during a conference, or the political ideology of someone we just met. Smooth social interaction necessitates keeping track of a variety of social data, such as individual attributes and interpersonal relationships and this referred to social working memory where both the thalamus and the insula are involved (Meyer and Lieberman, 2012). This illustrates our result in a sense that deficits in working memory in children, especially during learning-based activities, can result in children experiencing information overload and thus they may act out behaviorally or withdraw socially. In other words, this disrupted dFC between left AI and left thalamus, a finding that can be interpreted as support for the significant differences we found in IQ, working memory and behavioral problems between the two groups.

Importantly, we found a disrupted dFC between the right dAI and frontal middle gyrus. The implication of the frontal middle gyrus in competencies such as literacy, numeracy has been widely discussed by neuroimaging studies (Koyama et al., 2017). However, in line with our findings, Japee et al. (2015) suggested the role of middle frontal gyrus in the reorienting of attention which indicates the individuals' ability to efficiently pick and guide their attention toward behaviorally relevant information in their environment. Postcentral gyrus, on the other hand, was also involved in our study whereby dFC analysis showed alterations between this region and the right dAI. Intriguingly, Du et al. (2020) found a stronger within-network connectivity in the insula, the thalamus and the postcentral gyrus among other brain regions that constitute the so called punishment network. From a social psychological standpoint, this study strongly supports our results in such a way that people are compelled to comply out of fear of punishment since a minority position is aversive and it can result in hostility, condemnation, rejection from others, or social isolation. People may be encouraged to adhere to the majority position in order to escape such social penalty. Along with our results, the finding that the postcentral gyrus is amongst the potentially relevant brain areas in punishment processing, represents what could be a previously unknown function for this part of the brain and could provide a new target for researchers.

Our findings also showed altered dFC between left PI and right temporal pole. Generally, temporal areas comprising the temporal sulcus were found to be involved in the so called social attention and face perception particularly (Nummenmaa and Calder, 2009). Social attention refers to the social conduct that underpins joint attention, with the goal of coordinating attention allocation with others. These crucial social abilities are thought to be dependent on the development of attention skills such as (1) detecting eye-direction and (2) allocating attention to the same focus of attention as another human being. These cortical areas are critical in the processing of socially relevant cues such gaze following, eye direction, and head orientation (Hopkins et al., 2014). Conflicting with these findings, other literature interestingly suggested that temporal pole activations are more common in sophisticated emotional tasks like theory of mind activities, but are less common in simpler emotional tasks like emotional face perception or gaze perception tasks (Olson et al., 2007).

To sum up, by interpreting our results, we displayed the insula's ability to be both specialized and integrative and to operate both independently and cooperatively. This could explain how the insula functions as a network hub, coordinating input from various cognitive areas and activities. More replicated research on this area is required in the future especially for more specified aspects associated to social dysfunction such as social inattention.

### 4.1. Limitations

Power considerations may limit our ability to examine the consequences of medications and comorbidity with other diseases, particularly those with inattention impairments like autism, substance dependence, depression, anxiety or learning disorders. Furthermore, the consequences of head motion are in general a constant source of concern in in ADHD and imaging youth. To deal with the issue of motion, we omitted high-motion subjects as an exclusion criteria, and we suggest considering other methods such as regressing realignment parameters and performing individual-level separation techniques such as independent component analysis. Moreover, this research used cross-sectional data and merely served as a surrogate for maturational effects. Extending and refining dynamic connectivity techniques in ADHD will be possible in the future with larger and longitudinal subject populations.

Furthermore, in task-based fMRI trials, nothing is known about how dynamic functional connections are associated to social dysfunctioning. Although prior research has shown that the insula's static functional connections are altered within the salience and default mode networks (Zhao et al., 2017), there have been few studies that have looked at dynamic functional connections underlying social dysfunctioning during task states (Fong et al., 2019). Future research should look into how dynamic connections between insula subdivisions function in task-based fMRI studies.

Also, in our current configuration, functional connectivity evaluations were conducted using standardized insular sub-region seeds. Individual differences in the size and placement of functional areas may have an impact on connectivity maps. Individualized seeds created from a functional parcellation approach will aid future investigations in overcoming these methodological flaws.

Finally, It should be noted that the current dFC method is just one of several approaches for mapping the dynamic functional connections between distinct brain areas. Graph theoretical approaches (Braun et al., 2015), test statistics tracking time course variations (Zalesky et al., 2014), co-activation pattern identification (Chen et al., 2015), and employing time frequency information (Yang et al., 2014) have all held promise in identifying changes in functional connections that static FC methods fail to capture. Future research should look into how these different measurements can help to better understand the dynamic functional links of the insula subdivisions.

## 5. Conclusion

Clinicians can diagnose psychopathology associated to insular dysfunction and stratify differential remedies by translating basic science into clinically useful facts. To improve our treatments, we must connect the pieces of evidence to fully comprehend any brain region, learn how the brain works, and decode clinical manifestations. The regulation of instrumental parts of the brain, such as the insula, is at the heart of daily life's micro-operations. This study provides evidence that the ability of the insula to serve as a subjective experiencing and feeling center that combines emotional, sensory, cognitive, and motor functions is its primary purpose. Therefore, we suggest that the insula is implicated in social dysfunctioning in childern with ADHD, and hence, aberrant insular dFC and provides an essential connectivity marker associated with a diagnosis of ADHD.

## Data Availability Statement

The datasets presented in this article are not readily available due to privacy concerns, and access to the data used in this study is restricted. The data has been used only for research purposes and it does not contain any identifiable information. Requests to access the datasets should be directed to HZ, homerzeng@126.com.

## Ethics Statement

The studies involving human participants were reviewed and approved by the Medical Ethics Committee of Shenzhen Children's Hospital. Written informed consent to participate in this study was provided by the participants' legal guardian/next of kin.

## Author Contributions

AF, WH, and HZ are design of the work. TM, XW, YL, DF, XM, and LZ collected the data and organize the clinical information's. BY, AS, and HZ review the methods and whole manuscript. AF, WH, and DF analyze the data. AF writes the manuscript. Finally, all authors discussed the results and contributed to the final manuscript.

## Funding

This work was supported by a grant from Shenzhen Medical and Health Project (No. SZSM202011005).

## Conflict of Interest

The authors declare that the research was conducted in the absence of any commercial or financial relationships that could be construed as a potential conflict of interest.

## Publisher's Note

All claims expressed in this article are solely those of the authors and do not necessarily represent those of their affiliated organizations, or those of the publisher, the editors and the reviewers. Any product that may be evaluated in this article, or claim that may be made by its manufacturer, is not guaranteed or endorsed by the publisher.

## References

[B1] Ahmadi M. Kazemi K. Kuc K. Cybulska-Klosowicz A. Helfroush M. S. Aarabi A. (2021). Resting state dynamic functional connectivity in children with attention deficit/hyperactivity disorder. J. Neural Eng. 18, 0460d0461. 10.1088/1741-2552/ac16b3 34289458

[B2] Alloway T. P. Copello E. (2013). Working memory: The what, the why, and the how. Aust. Educ. Dev. Psychol. 30, 105–118. 10.1017/edp.2013.13

[B3] Association A. P.. (2013). “Dsm 5 diagnostic and statistical manual of mental disorders,” in DSM 5 Diagnostic and Statistical Manual of Mental Disorders (Washington, DC), 947.

[B4] Belfi A. M. Koscik T. R. Tranel D. (2015). Damage to the insula is associated with abnormal interpersonal trust. Neuropsychologia 71, 165–172. 10.1016/j.neuropsychologia.2015.04.003 25846668 PMC4417431

[B5] Bickart K. C. Dickerson B. C. Feldman Barrett L. (2014). The amygdala as a hub in brain networks that support social life. Neuropsychologia 63, 235–248. 10.1016/j.neuropsychologia.2014.08.013 25152530 PMC4981504

[B6] Biederman J. Faraone S. V. Spencer T. Wilens T. Norman D. Lapey K. A.. (1993). Patterns of psychiatric comorbidity, cognition, and psychosocial functioning in adults with attention deficit hyperactivity disorder. Am. J. Psychiatry 150, 1792–1798. 10.1176/ajp.150.12.1792 8238632

[B7] Braun U. Schäfer A. Walter H. Erk S. Romanczuk-Seiferth N. Haddad L.. (2015). Dynamic reconfiguration of frontal brain networks during executive cognition in humans. *Proc. Natl. Acad. Sci*. U.S.A. 112, 11678–11683. 10.1073/pnas.1422487112 26324898 PMC4577153

[B8] Broder J. Preston R. (2011). “Imaging the head and brain,” in Diagnostic Imaging Emerg. Physician (Saint Louis, MO: Elsevier), 1–45.

[B9] Brothers L.. (1990). The neural basis of primate social communication. Motiv. Emot. 14, 81–91. 10.1007/BF00991637

[B10] Buckholtz J. W. Asplund C. L. Dux P. E. Zald D. H. Gore J. C. Jones O. D.. (2008). The neural correlates of third-party punishment. Neuron 60, 930–940. 10.1016/j.neuron.2008.10.016 19081385

[B11] Bud Craig A. D.. (2009). How do you feel–now? The anterior insula and human awareness. Nat. Rev. Neurosci. 10, 59–70. 10.1038/nrn2555 19096369

[B12] Cereda C. Ghika J. Maeder P. Bogousslavsky J. (2002). Strokes restricted to the insular cortex. Neurology 59, 1950–1955. 10.1212/01.WNL.0000038905.75660.BD 12499489

[B13] Chee M. W. L. Soon C. S. Lee H. L. Pallier C. (2004). Left insula activation: a marker for language attainment in bilinguals. Proc. Natl. Acad. Sci. U.S.A. 101, 15265–15270. 10.1073/pnas.0403703101 15469927 PMC523445

[B14] Chen J. E. Chang C. Greicius M. D. Glover G. H. (2015). Introducing co-activation pattern metrics to quantify spontaneous brain network dynamics. Neuroimage 111, 476–488. 10.1016/j.neuroimage.2015.01.057 25662866 PMC4386757

[B15] Christiansen H. Hirsch O. Drechsler R. Wanderer S. Knospe E.-L. Günther T.. (2016). German validation of the conners 3u+ 00ae rating scales for parents, teachers, and children. Z. Kinder. Jugendpsychiatr. Psychother. 44, 139–147. 10.1024/1422-4917/a000408 27008903

[B16] Cieslik E. C. Mueller V. I. Eickhoff C. R. Langner R. Eickhoff S. B. (2015). Three key regions for supervisory attentional control: Evidence from neuroimaging meta-analyses. Neurosci. Biobehav. Rev. 48, 22–34. 10.1016/j.neubiorev.2014.11.003 25446951 PMC4272620

[B17] Conners K.. (2008). Conners 3rd Edn manual. New York, NY: Multi-health systems. Inc..[Google Scholar].

[B18] Davidson R. Ekman P. Saron C. Senulis J. Friesen W. (1990). Approach-withdrawal and cerebral asymmetry: emotional expression and brain physiology. J. Pers. Soc. Psychol. 58, 330–341. 10.1037/0022-3514.58.2.330 2319445

[B19] Deen B. Pitskel N. B. Pelphrey K. A. (2011). Three systems of insular functional connectivity identified with cluster analysis. Cereb. Cortex 21, 1498–1506. 10.1093/cercor/bhq186 21097516 PMC3116731

[B20] Du Y. Wang Y. Yu M. Tian X. Liu J. (2020). Resting-state functional connectivity of the punishment network associated with conformity. Front. Behav. Neurosci. 14, 617402. 10.3389/fnbeh.2020.617402 33390913 PMC7772235

[B21] Duffau H. Bauchet L. Lehéricy S. Capelle L. (2001). Functional compensation of the left dominant insula for language. Neuroreport 12, 2159–2163. 10.1097/00001756-200107200-00023 11447326

[B22] Fateh A. A. Cui Q. Duan X. Yang Y. Chen Y. Li D.. (2020). Disrupted dynamic functional connectivity in right amygdalar subregions differentiates bipolar disorder from major depressive disorder. Psychiatry Res. 304, 111149. 10.1016/j.pscychresns.2020.111149 32738725

[B23] Fong A. H. C. Yoo K. Rosenberg M. D. Zhang S. Li C.-S. R. Scheinost D.. (2019). Dynamic functional connectivity during task performance and rest predicts individual differences in attention across studies. Neuroimage 188, 14–25. 10.1016/j.neuroimage.2018.11.057 30521950 PMC6401236

[B24] Frith C. D. Frith U. (2006). The Neural basis of mentalizing. Neuron 50, 531–534. 10.1016/j.neuron.2006.05.001 16701204

[B25] Ghaziri J. Tucholka A. Girard G. Boucher O. Houde J.-C. Descoteaux M.. (2018). Subcortical structural connectivity of insular subregions. Sci. Rep. 8, 8596. 10.1038/s41598-018-26995-0 29872212 PMC5988839

[B26] Guo X. Yao D. Cao Q. Liu L. Zhao Q. Li H.. (2020). Shared and distinct resting functional connectivity in children and adults with attention-deficit/hyperactivity disorder. Transl. Psychiatry 10, 65. 10.1038/s41398-020-0740-y 32066697 PMC7026417

[B27] Harrington A.. (1995). “Unfinished business: models of laterality in the nineteenth century,” in Brain Asymmetry, eds R. J. Davidson and K. Hugdahl (Washington, DC: The MIT Press), 3–27.

[B28] Hodgetts S. Gallagher P. Stow D. Ferrier I. N. O'Brien J. T. (2017). The impact and measurement of social dysfunction in late-life depression: an evaluation of current methods with a focus on wearable technology. Int. J. Geriatr. Psychiatry 32, 247–255. 10.1002/gps.4632 27911019

[B29] Hopkins W. D. Misiura M. Reamer L. A. Schaeffer J. A. Mareno M. C. Schapiro S. J. (2014). Poor receptive joint attention skills are associated with atypical gray matter asymmetry in the posterior superior temporal gyrus of chimpanzees (Pan troglodytes). Front. Psychol. 5, 7. 10.3389/fpsyg.2014.00007 24523703 PMC3905213

[B30] Japee S. Holiday K. Satyshur M. D. Mukai I. Ungerleider L. G. (2015). A role of right middle frontal gyrus in reorienting of attention: a case study. Front. Syst. Neurosci. 9, 23. 10.3389/fnsys.2015.00023 25784862 PMC4347607

[B31] Jenkinson M. Bannister P. Brady M. Smith S. (2002). Improved optimization for the robust and accurate linear registration and motion correction of brain images. Neuroimage 17, 825–841. 10.1006/nimg.2002.1132 12377157

[B32] Kaufman J. Birmaher B. Brent D. Rao U. Flynn C. Moreci P.. (1997). schedule for affective disorders and schizophrenia for school-age children-present and lifetime version (K-SADS-PL): initial reliability and validity data. J. Am. Acad Child Adolesc. Psychiatry 36, 980–988. 10.1097/00004583-199707000-00021 9204677

[B33] Koyama M. S. O'Connor D. Shehzad Z. Milham M. P. (2017). Differential contributions of the middle frontal gyrus functional connectivity to literacy and numeracy. Sci. Rep. 7, 17548. 10.1038/s41598-017-17702-6 29235506 PMC5727510

[B34] Lahey B. B. Pelham W. E. Loney J. Lee S. S. Willcutt E. (2005). Instability of the DSM-IV subtypes of ADHD from preschool through elementary school. Arch. Gen. Psychiatry 62, 896. 10.1001/archpsyc.62.8.896 16061767

[B35] Lee T. M. Chan C. C. (2000). Stroop interference in chinese and english. J. Clin. Exp. Neuropsychol. 22, 465–471. 10.1076/1380-3395(200008)22:4;1-0;FT465 10923056

[B36] Leonardi N. Van De Ville D. (2015). On spurious and real fluctuations of dynamic functional connectivity during rest. Neuroimage 104, 430–436. 10.1016/j.neuroimage.2014.09.007 25234118

[B37] Li Y. Zhu Y. Nguchu B. A. Wang Y. Wang H. Qiu B.. (2020). Dynamic functional connectivity reveals abnormal variability and hyper-connected pattern in autism spectrum disorder. Autism. Res. 13, 230–243. 10.1002/aur.2212 31614075

[B38] Liao W. Li J. Duan X. Cui Q. Chen H. Chen H. (2018). Static and dynamic connectomics differentiate between depressed patients with and without suicidal ideation. Hum. Brain Mapp. 39, 4105–4118. 10.1002/hbm.24235 29962025 PMC6866497

[B39] Liao W. Wu G. R. Xu Q. Ji G. J. Zhang Z. Zang Y. F.. (2014). DynamicBC: a MATLAB toolbox for dynamic brain connectome analysis. Brain Connect. 4, 780–790. 10.1089/brain.2014.0253 25083734 PMC4268585

[B40] Lu B. Chen X. Li L. Shen Y. Chen N. Mei T.. (2018). Aberrant dynamics of spontaneous brain activity and its integration in patients with autism spectrum disorder. Chin. Sci. Bull. 63, 1452–1463. 10.1360/N972017-01260

[B41] Markostamou I. Rudolf J. Tsiptsios I. Kosmidis M. H. (2015). Impaired executive functioning after left anterior insular stroke: a case report. Neurocase 21, 148–153. 10.1080/13554794.2013.878725 25537237

[B42] Meyer M. L. Lieberman M. D. (2012). Social working memory: Neurocognitive networks and directions for future research. Front. Psychol. 3, 571. 10.3389/fpsyg.2012.00571 23267340 PMC3527735

[B43] Mills K. L. Bathula D. Dias T. G. C. Iyer S. P. Fenesy M. C. Musser E. D.. (2012). Altered cortico-striatal-thalamic connectivity in relation to spatial working memory capacity in children with ADHD. Front. Psychiatry 3, 2. 10.3389/fpsyt.2012.00002 22291667 PMC3265767

[B44] Nomi J. S. Farrant K. Damaraju E. Rachakonda S. Calhoun V. D. Uddin L. Q. (2016). Dynamic functional network connectivity reveals unique and overlapping profiles of insula subdivisions. Hum. Brain Mapp. 37, 1770–1787. 10.1002/hbm.23135 26880689 PMC4837017

[B45] Nomi J. S. Schettini E. Broce I. Dick A. S. Uddin L. Q. (2018). Structural connections of functionally defined human insular subdivisions. Cereb. Cortex 28, 3445–3456. 10.1093/cercor/bhx211 28968768 PMC6132280

[B46] Nummenmaa L. Calder A. J. (2009). Neural mechanisms of social attention. Trends Cogn. Sci. 13, 135–143. 10.1016/j.tics.2008.12.006 19223221

[B47] Olson I. R. Plotzker A. Ezzyat Y. (2007). The Enigmatic temporal pole: a review of findings on social and emotional processing. Brain 130, 1718–1731. 10.1093/brain/awm052 17392317

[B48] Patil A. U. Ghate S. Madathil D. Tzeng O. J. L. Huang H.-W. Huang C.-M. (2021). Static and dynamic functional connectivity supports the configuration of brain networks associated with creative cognition. Sci. Rep. 11, 165. 10.1038/s41598-020-80293-2 33420212 PMC7794287

[B49] Peelen M. V. Atkinson A. P. Vuilleumier P. (2010). Supramodal representations of perceived emotions in the human bbrain. J. Neurosci., 30, 10127–10134. 10.1523/JNEUROSCI.2161-10.2010 20668196 PMC6633378

[B50] Penfield W. Faulk M. E. (1955). The insula; further observations on its function. Brain 78, 445–470. 10.1093/brain/78.4.445 13293263

[B51] Rogers-Carter M. M. Christianson J. P. (2019). An insular view of the social decision-making network. Neurosci. Biobehav. Rev. 103, 119–132. 10.1016/j.neubiorev.2019.06.005 31194999 PMC6699879

[B52] Rohrer-Baumgartner N. Zeiner P. Egeland J. Gustavson K. Skogan A. Reichborn-Kjennerud T.. (2014). Does IQ influence associations between ADHD symptoms and other cognitive functions in young preschoolers? Behav Brai Funct. 10, 16. 10.1186/1744-9081-10-16 24884579 PMC4017812

[B53] Rubia K. Alegria A. Brinson H. (2014). Imaging the ADHD brain: disorder-specificity, medication effects and clinical translation. Expert. Rev. Neurother. 14, 519–538. 10.1586/14737175.2014.907526 24738703

[B54] Rubia K. Criaud M. Wulff M. Alegria A. Brinson H. Barker G.. (2019). Functional connectivity changes associated with fMRI neurofeedback of right inferior frontal cortex in adolescents with ADHD. Neuroimage 188, 43–58. 10.1016/j.neuroimage.2018.11.055 30513395 PMC6414400

[B55] Sayal K. Prasad V. Daley D. Ford T. Coghill D. (2018). ADHD in children and young people: prevalence, care pathways, and service provision. Lancet Psychiatry 5, 175–186. 10.1016/S2215-0366(17)30167-0 29033005

[B56] Scheinost D. Stoica T. Saksa J. Papademetris X. Constable R. T. Pittenger C.. (2013). Orbitofrontal cortex neurofeedback produces lasting changes in contamination anxiety and resting-state connectivity. Transl. Psychiatry 3, e250-e250. 10.1038/tp.2013.24 23632454 PMC3641411

[B57] Shappell H. M. Duffy K. A. Rosch K. S. Pekar J. J. Mostofsky S. H. Lindquist M. A.. (2021). Children with attention-deficit/hyperactivity disorder spend more time in hyperconnected network states and less time in segregated network states as revealed by dynamic connectivity analysis. Neuroimage 229, 117753. 10.1016/j.neuroimage.2021.117753 33454408 PMC7979530

[B58] Sonuga-Barke E. J. Castellanos F. X. (2007). Spontaneous attentional fluctuations in impaired states and pathological conditions: a neurobiological hypothesis. Neurosci. Biobehav. Rev. 31, 977–986. 10.1016/j.neubiorev.2007.02.005 17445893

[B59] Sun Y. Lan Z. Xue S.-W. Zhao L. Xiao Y. Kuai C.. (2021). Brain state-dependent dynamic functional connectivity patterns in attention-deficit/hyperactivity disorder. J. Psychiatr. Res. 138, 569–575. 10.1016/j.jpsychires.2021.05.010 33991995

[B60] Thomas R. Sanders S. Doust J. Beller E. Glasziou P. (2015). Prevalence of attention-deficit/hyperactivity disorder: a systematic review and meta-analysis. Pediatrics 135, e994-e1001. 10.1542/peds.2014-3482 25733754

[B61] Touroutoglou A. Hollenbeck M. Dickerson B. C. Feldman Barrett L. (2012). Dissociable large-scale networks anchored in the right anterior insula subserve affective experience and attention. Neuroimage 60, 1947. 10.1016/j.neuroimage.2012.02.012 22361166 PMC3345941

[B62] Usami M.. (2016). Functional consequences of attention-deficit hyperactivity disorder on children and their families. Psychiatry Clin. Neurosci. 70, 303–317. 10.1111/pcn.12393 27061213

[B63] Varjačić A. Mantini D. Levenstein J. Slavkova E. D. Demeyere N. Gillebert C. R. (2018). The role of left insula in executive set-switching: lesion evidence from an acute stroke cohort. Cortex 107, 92–101. 10.1016/j.cortex.2017.11.009 29248158 PMC6181803

[B64] Wang M. Hu Z. Liu L. Li H. Qian Q. Niu H. (2020). Disrupted functional brain connectivity networks in children with attention-deficit/hyperactivity disorder: evidence from resting-state functional near-infrared spectroscopy. Neurophotonics 7, 1. 10.1117/1.NPh.7.1.015012 32206679 PMC7064804

[B65] Yan C.-G. Wang X.-D. Zuo X.-N. Zang Y.-F. (2016). Dpabi: data processing &analysis for (resting-state) brain imaging. Neuroinformatics 14, 339–351. 10.1007/s12021-016-9299-4 27075850

[B66] Yang Y. Yang B. Zhang L. Peng G. Fang D. (2021). Dynamic functional connectivity reveals abnormal variability in the amygdala subregions of children with attention-deficit/hyperactivity disorder. Front. Neurosci. 15, 648143. 10.3389/fnins.2021.648143 34658751 PMC8514188

[B67] Yang Z. Craddock R. C. Margulies D. S. Yan C.-G. Milham M. P. (2014). Common intrinsic connectivity states among posteromedial cortex subdivisions: Insights from analysis of temporal dynamics. Neuroimage 93, 124–137. 10.1016/j.neuroimage.2014.02.014 24560717 PMC4010223

[B68] Zalesky A. Fornito A. Cocchi L. Gollo L. L. Breakspear M. (2014). Time-resolved resting-state brain networks. Proc. Natl. Acad. Sci. U.S.A. 111, 10341–10346. 10.1073/pnas.1400181111 24982140 PMC4104861

[B69] Zhao Q. Li H. Yu X. Huang F. Wang Y. Liu L.. (2017). Abnormal resting-state functional connectivity of insular subregions and disrupted correlation with working memory in adults with attention deficit/hyperactivity disorder. Front. Psychiatry 8, 200. 10.3389/fpsyt.2017.00200 29075206 PMC5641567

