# Supplementary figures and images for: Abnormal Insular Dynamic Functional Connectivity and Its Relation to Social Dysfunctioning in Children With Attention Deficit/Hyperactivity Disorder
Source: Front Neurosci. 2022 May 31;16:890596. doi: 10.3389/fnins.2022.890596 (PMC9197452; doi:10.3389/fnins.2022.890596)

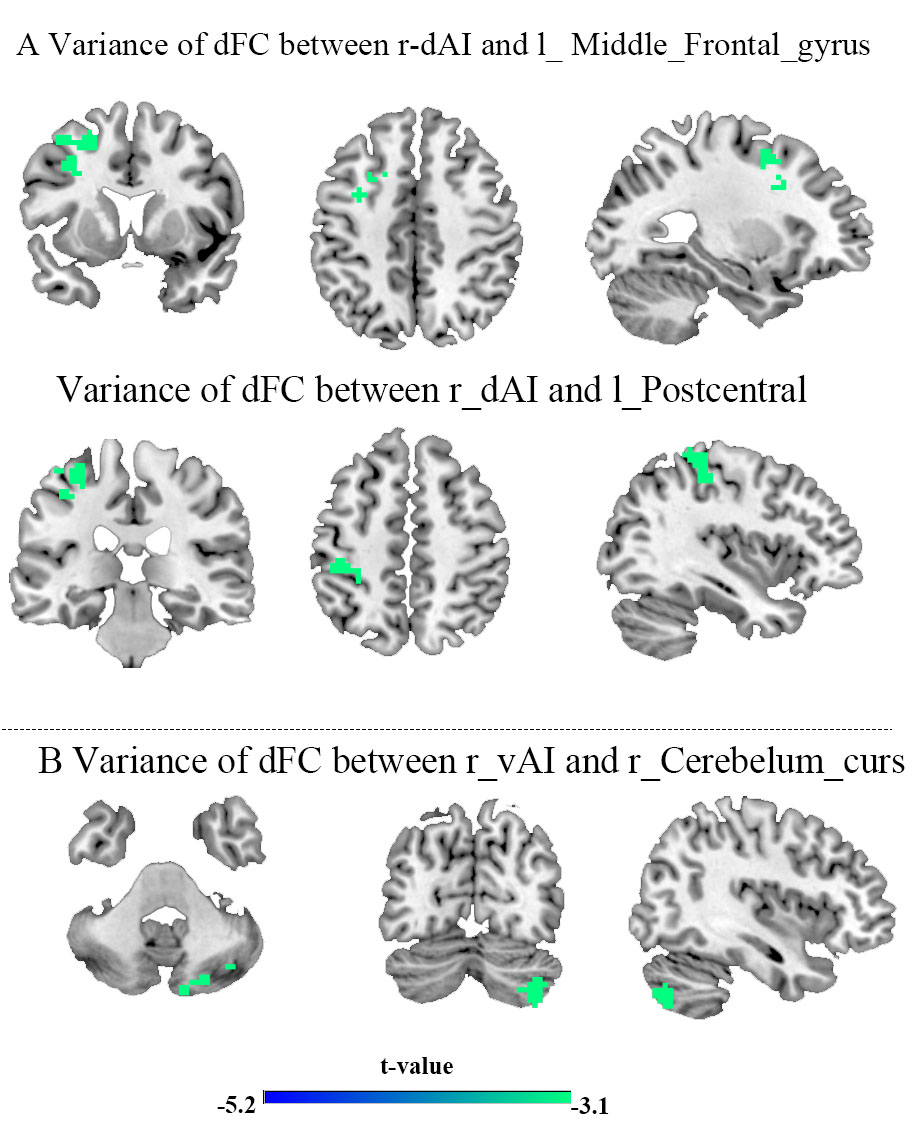

Supplement: Supplementary file 1 [file Image_1.JPEG]

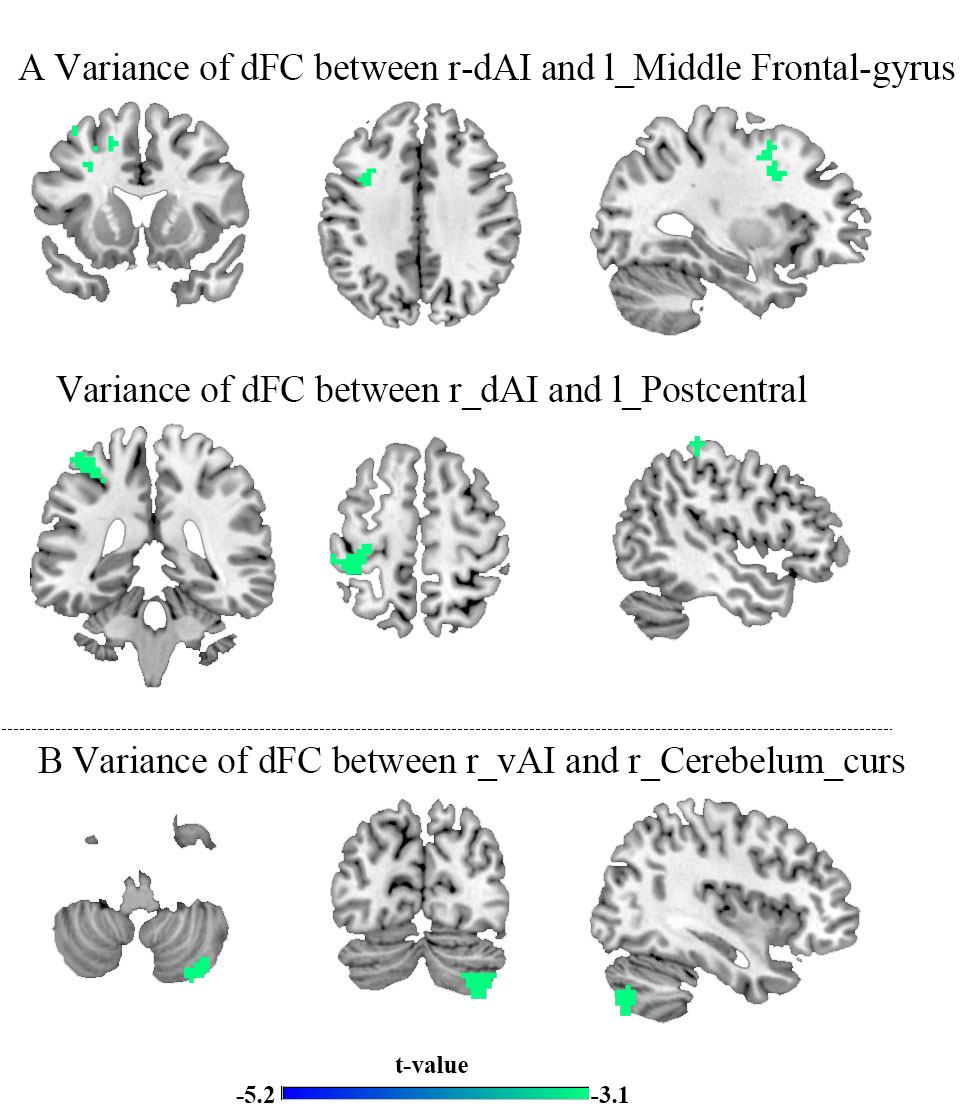

Supplement: Supplementary file 2 [file Image_2.JPEG]

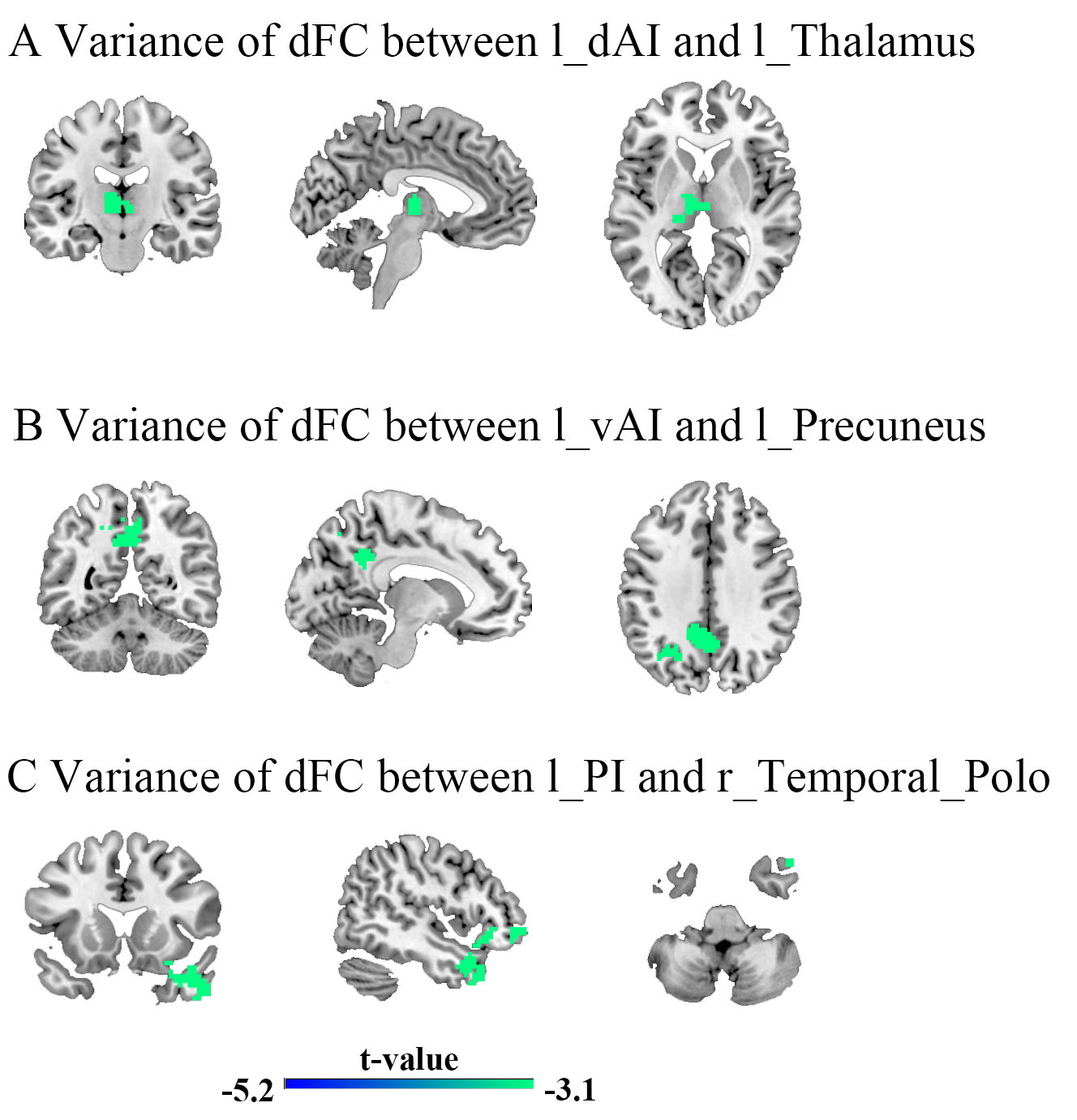

Supplement: Supplementary file 3 [file Image_3.JPEG]

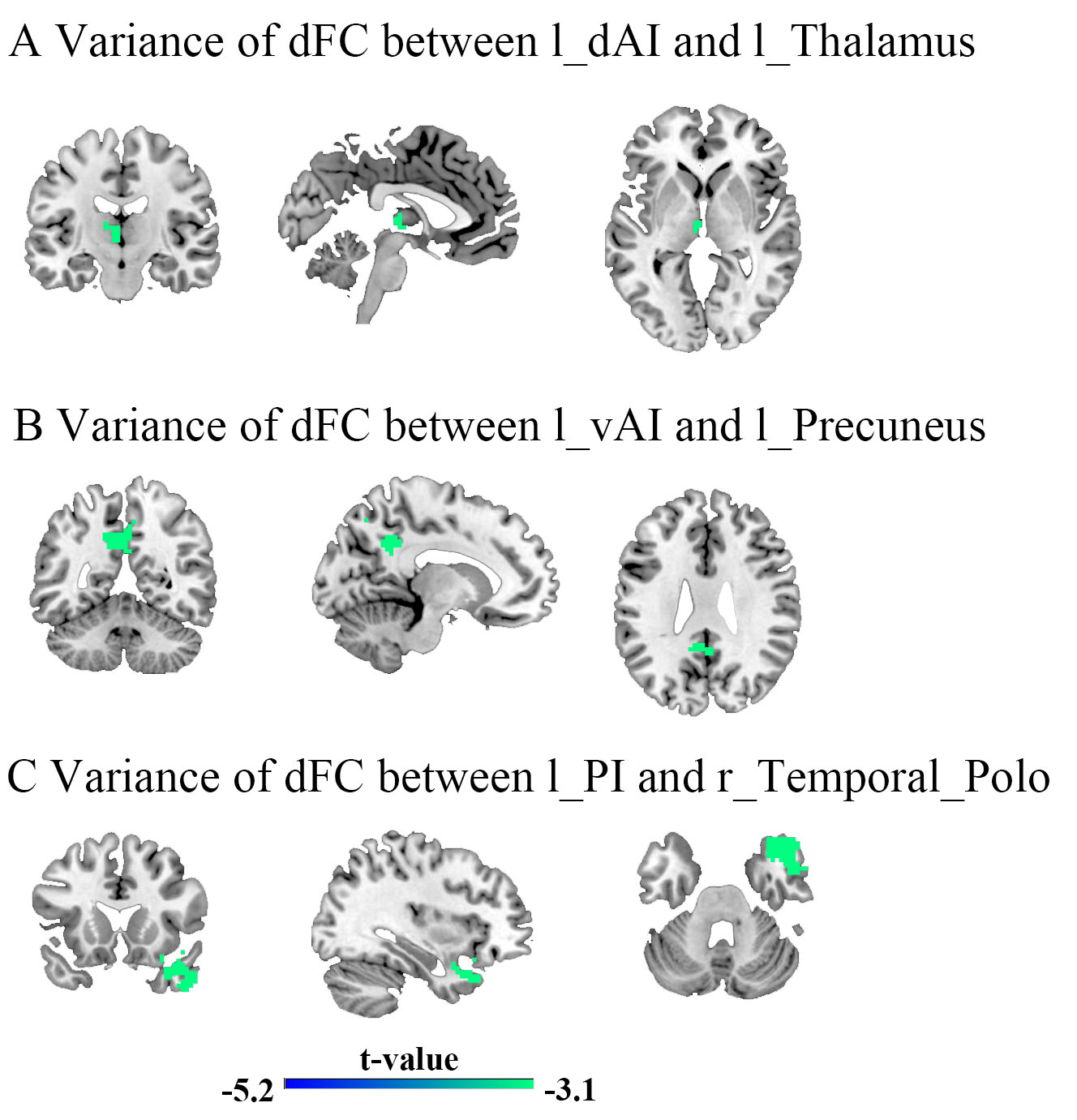

Supplement: Supplementary file 4 [file Image_4.JPEG]
